# Estimation of menstrual blood loss volume based on menstrual diary and laboratory data

**DOI:** 10.1186/1472-6874-12-24

**Published:** 2012-08-20

**Authors:** Ulrike Schumacher, Jens Schumacher, Uwe Mellinger, Christoph Gerlinger, Andreas Wienke, Jan Endrikat

**Affiliations:** 1 Medical Affairs Support, Jenapharm GmbH und Co KG 07743, Jena, Germany; 2 Center for Clinical Studies, Universitätsklinikum Jena, 07747 Jena, Germany; 3 Institute of Stochastics, University of Jena, 07737, Jena, Germany; 4 Global Clinical Statistics, Bayer Pharma AG, 13342, Berlin, Germany; 5 Institute for Medical Epidemiology, Biometrics and Computer Science, Universitätsklinikum Halle, 06112, Halle, Germany; 6 Global Medical Affairs, Bayer Pharma AG, 13342, Berlin, Germany; 7 Department of OBGYN, Saarland University, 66421, Homburg/Saar, Germany

## Abstract

**Background:**

Abnormal uterine bleeding is often investigated in clinical studies and critical to identify during gynecological consultation. The current standard for quantification of menstrual blood loss is the alkaline-hematin-method. However, this method is expensive and inconvenient for patients. Bleeding diaries, although widely used, provide only qualitative information on menstrual blood loss. Other methods have been developed, but still do not provide reliable quantitative data.

**Methods:**

We estimated blood loss volume using data from two clinical studies in women suffering abnormal menstrual bleeding. These estimations were derived from mixed linear models based on diary data, hematological parameters and age. To validate the models, we applied our results to data from a third study with a similar patient population.

**Results:**

The resulting best fitting model uses diary entries on bleeding intensity at a particular day, information on occurrence and frequency of single bleeding intensities in defined time windows, hemoglobin and ferritin values and age of the patient all as predictors of menstrual blood loss volume. Sensitivity and specificity for the diagnosis of excessive bleeding were 87% and 70%, respectively. Our model-based estimates reflect the subjective assessment by physicians and patients in the same way as the measured values do.

When applying the model to an independent study, we found a correlation of 0.73 between estimated and measured values for the blood loss in a single day. Further models with reduced number of parameters (simplified for easier practical use) still showed correlation values between 0.69 and 0.73.

**Conclusions:**

We present a method for estimating menstrual blood loss volume in women suffering from prolonged or excessive menstrual bleeding. Our statistical model includes entries from bleeding diaries, laboratory parameters and age and produces results which correlate well with data derived by the alkaline-hematin-method. Therefore, this model may be used to estimate menstrual blood loss volume in both routine gynecological counseling and clinical studies.

## Background

Abnormal uterine bleeding is an important clinical topic both within clinical studies and during gynecological consultation [1-3]. Changes in bleeding intensity or intracyclic bleeding might be symptoms of pathological processes. Dysfunctional uterine bleedings, including heavy, frequent and prolonged bleedings may not only substantially decrease quality of life, but can also cause iron-deficiency anemia [4,5]. A menstrual diary is the most commonly used tool to monitor menstrual blood loss, and the WHO has defined the main principles used to evaluate vaginal bleedings from diaries [6]. To supplement these principles, Gerlinger et al. have proposed new standards for diary evaluation [7,8].

However, single bleeding episodes remain incommensurable and menstrual blood loss volume (MBLV) is difficult to estimate based on diaries. The current standard for quantification of menstrual blood loss is the alkaline-hematin-method [9], for which women need to collect and date all sanitary protection items for laboratory analysis. Recently, the method was extended to a semi-automated procedure and validated for towels containing ultra-absorbing materials [10]. However, this method remains both expensive and inconvenient.

Methods based on direct measurement of sanitary item weight change [11,12] are inherently imprecise because blood fraction accounts for only about 30 – 40% of menstrual fluid and this portion is highly variable between individuals [13,14].

Semi-quantitative assessment of blood loss by comparing sanitary item stain with given diagrams (Pictorial Blood Loss Assessment Chart, PBAC) [15] is another method to assess blood loss. Using an appropriate cutoff point, Janssen et al. reported predictive values with respect to menorrhagia up to 85.9% for positive tests and 84.9 for negative tests [16]. Unfortunately reproducibility of results of PBAC was challenged [17]. Nonetheless, a recent study found that PBAC is a simple and accurate tool for semi-objective measurement of menstrual blood loss [18]. The correlation between pictorial assessment results and values measured by the alkaline-hematin-method is quite good, however the amount of blood not captured by sanitary items is considerable [19]. Other bleeding characteristics such as the number of sanitary items used highly depend on the socio-economic status and the individual hygienic needs of women [20].

We think that a tool to estimate menstrual blood loss based on diaries and additional routine laboratory parameters would be very helpful. A first comparison of MBLV as measured by alkaline-hematin-method with diary entries in women with menorrhagia by Fraser et al. [21] yielded mean values for the different categories. However, individual ranges were wide and Fraser et al. concluded that only alkaline hematin extraction provides reliable assessments of menstrual blood loss volume. However, these estimates were based solely on arithmetic means and did not account for patient-specific differences in menstrual diary entries. The perception of menstrual blood loss is highly subjective. The idea of the present analysis was to derive information on the woman’s individual “assessment range” using additional parameters such as hematological values (hemoglobin, ferritin) which are routinely measured in clinical studies, other diary characteristics (e.g. number of days with the different intensities), age and possibly other influencing factors.

In order to develop a statistical model that provides improved estimation of MBLV based on menstruation diaries and accounting for additional parameters, we used data from three studies on treatment of dysfunctional uterine bleeding.

## Methods

### Study design

We retrospectively analyzed diary data on bleeding intensities from two prospective studies investigating treatment of dysfunctional uterine bleeding with an oral contraceptive containing estradiol valerate/dienogest in order to derive estimates of MBLV. Details on the study results have been published earlier: Study 1: [22], Study 2: [23].

A third prospective study on treatment of idiopathic menorrhagia with a levonorgestrel containing intrauterine device (IUS) versus Medroxyprogesterone acetate retrospectively served to validate our findings. Details on the third study have been published elsewhere [24].

All studies were conducted in accordance with all local legal and regulatory requirements and with the ethical principles that have their origin in the Declaration of Helsinki and the ICH-GCP Guidelines. The protocols and amendments were reviewed and approved by each site’s Independent Ethics Committee (IEC) or Institutional Review Board (IRB) before the start of the studies and before implementation of the amendments.

All three studies had a similar design. Patients received bleeding diaries to record daily bleeding intensity (BI) in four categories: SPOTTING and LIGHT, NORMAL or HEAVY bleeding. Days without bleeding and spotting were marked with „no bleeding“. On days with bleeding or spotting, sanitary items were collected on a daily basis. Daily MBLV was determined by applying the alkaline-hematin-method [9,25].

In addition, hemoglobin, hematocrit and serum ferritin were measured with standard methods in local laboratories. Blood samples were taken at several time points during the studies. Intermediate blood values have been calculated by linear interpolation.

Age was recorded at time of study inclusion.

A bleeding episode was defined as according to Gerlinger et al. [8] as a period of days with bleeding or spotting preceded and followed by at least 2 bleed-free days.

### Study population

All three studies included patients with menstrual bleeding abnormalities. The first two studies investigated women with dysfunctional uterine bleeding defined as excessive (at least two episodes with a total blood loss of at least 80 ml MBLV during the reference period of 90 days), frequent (more than 5 episodes with a total of at least 20 bleeding days during the reference period), or prolonged (at least 2 episodes of 8 or more days each during the reference period). The third study, the validation study, included patients with idiopathic menorrhagia, defined as a total blood loss of at least 80 ml during at least two of three reference cycles. We used all daily patient data, where information on BI, MBLV, hematological values and age was available, even of screening failures who were not included in the original studies analyses.

An overview of demographic data is given in Table 1.

**Table 1:** Summary of demographic data of the three study populations

	**Study 1**	**Study 2**	**Validation study 3**
Number of patients used	190	250	162
Number of days	9452	12431	3242
Mean age (± standard deviation) [years]	36.9 (± 7.1)	39.2 (± 6.9)	38.8 (± 5.3)
Median cycle length (range) [days]	28 (9 – 38)	28 (14 – 38)	28 (7 – 33)
Mean duration [days] of bleeding episodes (range)	7.6 (4 – 60)	6.9 (3 – 17)	6.3 (3 – 14)

### Statistical analysis

Since the distribution of measured MBLV values was highly skewed, all analyses were performed after logarithmic transformation of MBLV values. Transformed values were approximately normally distributed but with remarkable differences in variability between categories. This was accounted for in the model by category-specific variances.

In addition to the BI categories, we considered three main groups of predictors for MBLV:

Hematological values (hemoglobin, hematocrit and serum ferritin)

Additional diary information apart from BI, i.e., frequency of the BI SPOTTING, LIGHT, NORMAL and HEAVY during several defined time windows

Age of the patient.

Inclusion of these additional predictors can be interpreted as an attempt to quantify subjective, patient-specific differences in “assessment ranges”. As this can only partly be achieved, we used a mixed model approach to account for remaining patient-specific effects (i.e. consistent inter-individual differences in the assignment of blood loss to categories of bleeding intensity). Our basic model therefore is a linear mixed model of the form

(1) $$y_{\mathrm{ik}}=\beta_{1}+\beta_{2}x_{2,ik}+\beta_{3}x_{3,ik}+\ldots +\beta_{p}x_{p,ik}+\gamma_{i}+\varepsilon_{\mathrm{ik}}$$

where $y_{\mathrm{ik}}$ is the MBLV of the *i*-th patient on day *k*, *β*_*l*_ are the regression parameters to be estimated, $x_{l,ik}$ are the respective covariates of patient *i* at day *k* , $\gamma_{i}$ denotes a random effect associated with patient *i,* assumed to be normally distributed, and $\varepsilon_{\mathrm{ik}}$is the residual error of patient *i* at day *k,* assumed to be normally distributed with category-specific variance.

Because subjective threshold values may also lead to patient-specific differences between categories we additionally included a random patient * BI interaction term.

Model selection proceeded sequentially: Starting with a model based on BI only, models including different combinations of laboratory parameters with and without interaction with BI have been examined, resulting in the best hematological model.

In a second step, we considered diary-derived parameters

occurrence of each single BI in the current bleeding episode,

occurrence of each single BI in the entire diary, and

number of days reporting each single BI during several time windows

Possible time frames of interest considered were:

the actual bleeding episode comprising the day of interest

the preceding episode

all bleeding episodes during a time period most probably containing the last menstrual/withdrawal bleeding episode, here assessed as „all episodes extending at least 1 day into the 30 days preceding the actual episode“

a baseline episode which reflects the inclusion criterion of the studies, defined here as the second heaviest episode during baseline. Heaviest was defined according to Fraser et al. [21] by using the means for MBLV per BI given there as a rough estimation.

In this step we initially searched for the best diary-parameter / time-frame combination separately for each single BI and subsequently considered combinations of those models incorporating information about several BI.

In the third step we added age to the model.

However, since the best model (M1, see results) contains several parameters probably not always available, we considered in addition a number of reduced models for more practical use.

(M2) accounts for 1) number of days with heavy bleeding, 2) occurrence of normal bleeding, 3) occurrence of light bleeding and 4) occurrence of spotting. All parameters were assessed with respect to the actual bleeding episode. Furthermore, we included 5) hemoglobin (Hb), 6) serum-ferritin (Fe, including interaction with BI) and 7) age.

(M3) accounts for the laboratory parameters only as given in (M1),

(M4) accounts for the diary parameters only as given in (M1),

(M5) accounts for age only.

All models include the BI of the respective bleeding day.

All models were analyzed on the basis of data from study 1 and 2 and were compared using Bayes’ Information Criterion, BIC [26]. Here smaller values of this criterion indicate more adequate statistical models. Computations were performed using SAS software package (SAS Institute Inc., Cary, NC, USA) version 9.1.3 under Windows (Version 5.1).

We validated our models by using the data of study 3. For evaluation of reproducibility, we calculated the Spearman correlation coefficient between estimated MBLV and measured MBLV. For evaluation of medical relevance, we compared change of blood loss - estimated and measured MBLV - with the patient’s efficacy assessment by using the Global Improvement Item of the Clinical Global Impression Score (CGI) [27].

For practical use, assessment of bleeding episodes with excessive bleeding is of major clinical interest. Therefore, sensitivity and specificity of assessment via estimated MBLV versus assessment via measured MBLV values as gold standard were evaluated.

## Results

For laboratory parameters, the best model (M1) includes

hemoglobin and

serum-ferritin including BI interaction.

The inclusion of hematocrit did not lead to improvement of the model.

Evaluation of diary parameters leads to the best model containing:

number of days with HEAVY bleeding in the current bleeding episode

number of days with HEAVY bleeding in the preceding bleeding episode

occurrence of HEAVY bleeding in the entire diary

mean number of days with NORMAL bleeding during all episodes reaching into the last 30 days before current episode

number of days with NORMAL bleeding in the second heaviest baseline-episode

number of days with LIGHT bleeding in the second heaviest baseline-episode

occurrence of LIGHT bleeding in the current episode

occurrence of SPOTTING in the current episode

Examination of the

age of the patient

resulted in a model including also interaction of age with BI.

Permutation of implementation of these three blocks of parameters confirmed necessity of all. Table 2 gives values of BIC for the best and the reduced models. Estimates and their standard errors for models (M1) to (M5) are listed in the Additional file 1.

**Table 2:** Comparison of models

**Model**	**Bayes’ Information Criterion (BIC)**	**Likelihood ratio test (with respect to initial model)**		
		**Likelihood ratio**	**Degrees of freedom**	**p value**
Initial model based solely on BI	57861.7	-	-	-
(M1)	55740.0	2514.4	42	<0.001
(M2)	55781.7	2379.8	30	<0.001
(M3)	55837.6	2114.0	9	<0.001
(M4)	55881.3	2290.2	31	<0.001
(M5)	55925.1	2016.5	8	<0.001

The BIC values of the different models clearly show the advantage of model (M1). Each further simplification (M2) – (M5) results in worse model indicated by an increase of the BIC. However, all models have a very clear advantage with respect to the model without any supportive parameters which is also confirmed by likelihood ratio tests comparing (M1) – (M5) to the initial model.

### Validation

For the validation study, we compared estimated values based on model (M1), given as mean of estimated MBLV values (geometric mean / median), with values measured by means of alkaline-hematin-method (measured values). The estimated MBLV value for days with spotting was 3.23 ml (2.19 ml / 3.16 ml); while the measured value was 3.04 ml (1.65 ml / 1.10 ml). For days with light bleeding, the estimated value was 7.52 ml (6.36 ml / 7.27 ml) and the measured value was 9.23 ml (4.70 ml / 4.00 ml). We estimated blood loss on days with normal bleeding as 31.57 ml (29.36 ml / 30.05 ml) and measured blood loss as 30.39 ml (18.21 ml / 22.10 ml). On days with heavy bleeding the estimated value was 70.25 ml (67.44 ml / 68.42 ml), whereas the measured figure was 60.86 ml (48.01 ml/ 51.20 ml) in the validation study. The Spearman correlation coefficient (standard error) between single estimated and measured values was 0.73 (0.012) with p < 0.0001. When comparing complete episodes, as usually done in studies, the Spearman correlation between estimated and measured MBLV sums was 0.62 (0.031) with p < 0.0001.

Application of model (M2) resulted in only slightly changed estimates. Spearman correlation remains 0.73 (0.012) with p < 0.0001 for day-wise comparison and 0.64 (0.030) with p < 0.0001 for the episodes. Even with the most simple model (M5), the correlation between day-wise estimates and measurements was 0.69 (0.012) (Table 3).

**Table 3:** Spearman correlation between estimated and measured MBLV in validation study

**Model**	**Day-wise correlation (s.e.)**	**Episode-wise correlation (s.e.)**
(M1)	0.73 (0.012)	0.62 (0.031)
(M2)	0.73 (0.012)	0.64 (0.030)
(M3)	0.72 (0.012)	0.60 (0.032)
(M4)	0.70 (0.013)	0.54 (0.033)
(M5)	0.69 (0.012)	0.58 (0.032)

(s.e. = standard error).

**Figure 1:**
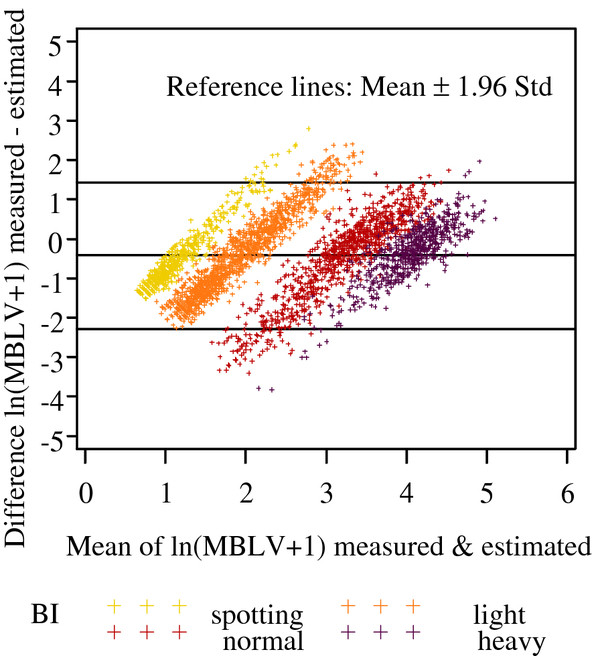
Validation study, mean and difference of measured and estimated MBLV values.

A Bland-Altman-Plot [28] relating the difference between measured and estimated values to the mean of both quite well illustrated the results (display of log-transformed values). Within individual categories values were equalized, low values were overestimated and high values underestimated. Generally, estimated values were slightly higher than measured. There was a clear separation between SPOTTING and LIGHT bleeding on the one hand and NORMAL and HEAVY bleeding on the other hand (Figure 1). 

Both, estimated and measured MBLV values, were used to predict patients and investigators subjective impression of treatment effect [27], assessed as change from a baseline phase (56 days pretreatment) to final phase (last 56 days under treatment). Estimated and measured values were almost equivalent and showed similar correlation with the patient’s subjective impressions (Table 4). 

**Table 4:** Comparison of change in bleeding amount with Clinical Global Impression scale

	**Mean change (± standard deviation)**	**Association with investigators assessment, Spearman correlation (s.e.) n = 111**	**Association with patients assessment, Spearman correlation (s.e.) n = 111**
Estimated MBLV, by day	−9.02 ± 16.32	0.48 (0.084)	0.44 (0.086)
Measured MBLV, by day	−11.79 ± 20.51	0.42 (0.087)	0.43 (0.086)

(s.e. = standard error).

In the validation study, a total of 648 episodes were available and could be used to investigate the classification sensitivity and specificity for excessive bleeding episodes.

When used for classification of bleeding episodes as excessive, our estimation identified 380 of 435 excessive episodes (according to the definition based on measured MBLV), corresponding to a sensitivity of 87%. Of the 213 not excessive episodes (based on measured MBLV) 149 were classified as not excessive based on the estimated MBLV as well. This provides a specificity of 70% (see Table 5).

**Table 5:** Comparison of assessment of bleeding episodes as excessive

**Classification according estimated MBLV**	**Classification according measured MBLV**		**Total**
	**Excessive**	**Not excessive**	
excessive	380 (58.6%)	64 (9.9%)	444 (68.5%)
	87.4% of excessive	30.0% of not excessive	
not excessive	55 (8.5%)	149 (23.0%)	204 (31.5%)
	12.6% of excessive	69.9% of not excessive	
Total	435 (67.1%)	213 (32.9%)	648 (100%)

(n = 648 episodes; excessive means Sum MBLV of episode > 80 ml).

## Discussion

Assessment of menstrual blood loss is a ubiquitous topic in the context of clinical studies that investigate effects of drugs and devices on menstrual bleeding pattern as well as in routine gynecological counseling. The current method of choice is the alkaline-hematin-method [9]. Although this method sees broad use and is the exactest method available, it is cumbersome, inconvenient and expensive. On the other hand, menstruation diaries, although widely used, do not provide sufficient quantitative resolution to compare MBLV of different bleeding episodes. Semi-quantitative assessment methods like the PBAC [15] show good sensitivity and specifity for menorrhagia with respect to the alkaline-hematin-method [16]. Unfortunately they are not completely precise, in particular with respect to blood lost outside sanitary items.

Using bleeding intensity as assessed by categories and available from diaries, we show here that a model-based statistical estimate of menstrual blood loss can be derived. By including laboratory parameters (hemoglobin and serum ferritin) and age we present here simple and accurate methods for estimation of MBLV.

Estimated MBLV values determined by our model showed a remarkable correlation of 0.73 to measured values, when we validated the model using a third study. A sensitivity of 87.4% and a specificity of 69.9% for assessment of excessive bleedings are sufficient for clinical use.

Beside the bleeding intensity, the following auxiliary parameters provide valuable information:

### Hematological parameters

Low hemoglobin values and ferritin values can be indicators of substantial menstrual blood loss, sometimes leading to adaptation and hence higher thresholds for the bleeding categories. The estimates especially for days with a blood loss more than SPOTTING increase with decreasing hemoglobin and ferritin, in agreement to the findings of Hallberg et al. [5].

### Number of days with the certain BIs and occurrence of the single BIs in defined time windows

In general, the frequency of the single BIs in the diary gives some insight into the woman’s individual assessment behavior. Extensive usage of the category “HEAVY” in a given episode can be a sign for having a lower personal threshold for this BI. On the other hand, if a woman has had many days of heavy bleeding in her menstrual history, this may result in increasing personal thresholds for MBLV classified as heavy. This may be due to acclimatization to a certain amount of blood loss.

In the event that a single BI is completely missing in an episode or in the whole diary, a typical effect can be observed: Here the other BIs tend to fill in the gap. If HEAVY is not used at all, all other BIs correspond to a higher level of blood loss, if SPOTTING is missing, the values of LIGHT, but also NORMAL decrease, and if LIGHT is absent, SPOTTING values rise and NORMAL and HEAVY decrease.

### Age

Hallberg et al. and Fraser et al. [5,21] reported an increasing MBLV with increasing age. Consequently, women get familiar with their MBLV and the estimates for the single BIs increase with age.

Modeling the MBLV based on diary BI readings showed, that the BIs are not equally spaced, on either a direct or logarithmic scale. Rather there is a more clear separation between LIGHT and NORMAL bleeding than within the pairs SPOTTING-LIGHT and NORMAL-HEAVY, respectively.

Fraser et al. [21] emphasizes that the values of MBLV scatter notably within the single BI and that these values might even be contradictory. In agreement with this observation, we also see that, as in every large data pool, implausible values occur. Beside false values, which might be due to entry errors, a considerable segment of variability is due to individual differences. We were able to reduce this segment of variability by including additional information on the bleeding behavior, laboratory parameters and age, finding a high correlation between estimated and observed MBLV. It is important to note that a certain amount of menstrual fluid is lost for MBLV measurement. Women lose blood not captured by sanitary items when changing them, or when clothes are soaked [19,29,30]. Therefore, MBLV measurement as determined by the alkaline-hematin-method might not reflect actual MBLV. However, women perceive this amount of blood and are able to account for it in their diary. With this in mind, a menstruation diary could be a better descriptor of blood loss, a possibility which is reflected by the slightly higher correlations between estimated values and patients/investigators assessment compared to the correlation between measured values and patients/investigators assessment (Table 4). Given a large number of days and women, single events of a larger fraction of ‘lost’ blood average out.

### Limitations

As a statistical model, our method does not provide precise blood loss measurement. In studies where MBLV is the primary efficacy outcome, the alkaline-hematin-method will remain the standard, although that method does not account for blood loss outside sanitary protection. Moreover, while we only tested our model in a single trial, further studies will be needed to verify reproducibility of this model.

Another limitation of our work is the narrowly defined patient population. Only patients with baseline bleeding problems like excessive, frequent or prolonged bleeding have been included. Similar analyses for healthy subjects will still need to be performed. Nevertheless, since MBLV is of special interest in patients complaining about bleeding problems, our results remain clinically meaningful.

Explicit estimation of patient specific effects should be possible (e.g., from baseline data) and would also be of clinical interest. However, the approach to estimate the random patient effects turned out to be inappropriate for the kind of studies available, because patients had different bleeding amounts at baseline and under treatment. So a number of women exclusively used HEAVY and NORMAL BI at baseline, but mostly and sometimes exclusively LIGHT and SPOTTING under treatment, foiling any estimation. Examination of patient specific effects should thus be performed in a healthy population or minimally in patients with stable bleeding patterns.

A number of additional parameters influencing assessment of BI (e.g. hormone levels / ovulation [31]) were not evaluated in this analysis.

## Conclusions

We propose a statistical model for estimating MBLV in women suffering from prolonged or excessive menstrual blood loss. The model encompasses entries from bleeding diaries, laboratory parameters and age. The results correlated well with data derived by the alkaline-hematin-method and can be used to estimate MBLV in clinical studies and during routine gynecological counseling.

## Competing interests

US is a part-time employee of Jenapharm GmbH & Co. KG. UM, CG, and JE are full-time employees of the Bayer Pharma AG. JS and AW have no financial or non-financial competing interests.

## Authors’ contributions

US was involved in the conception of this evaluation, in the analysis and interpretation of data, and in development and review of the manuscript for intellectual content. JS was involved in the conception of this evaluation and in development and review of the manuscript for intellectual content. UM was involved in selection of suitable studies contributing data for the analysis, in the evaluation of the underlying studies and in review of the manuscript for intellectual content. CG was involved in the conception of this evaluation and in review of the manuscript for intellectual content. AW was involved in conception and supervising of the modeling process, and in review of the manuscript for intellectual content. JE was involved in the medical interpretation of the results and drafting the manuscript. All authors read and approved the final manuscript.

## Pre-publication history

The pre-publication history for this paper can be accessed here:

http://www.biomedcentral.com/1472-6874/12/24/prepub

## Supplementary Material

Additional file 1 Appendix Tables. Click here for file
