# Supplementary material for: Estimation of menstrual blood loss volume based on menstrual diary and laboratory data
Source: BMC Womens Health. 2012 Aug 20;12:24. doi: 10.1186/1472-6874-12-24 (PMC3554478; doi:10.1186/1472-6874-12-24)
Supplement: Additional file 1 — Appendix Tables. [file 1472-6874-12-24-S1.pdf]

Appendix: Tables

Model (M1): Evaluation of blood loss

Let

Hb_{ik}	Hemoglobin [g/dl] of patient i at day k (imputed by linear interpolation)
Fe_{ik}	Serum-ferritin [ng/ml] of patient i at day k, (imputed by linear interpolation)
H_{ik}	Number of days with HEAVY bleeding during the episode of interest of patient i with respect to day k
H_{ik}^{-1}	Number of days with HEAVY bleeding during the preceding episode of patient i with respect to day k
H_{i}^{I}	Occurrence [0=no, 1=yes] of HEAVY bleeding during the complete available diary of patient i
N_{ik}^{MW}	Mean number of days with NORMAL bleeding of all episodes of the last 30 days of patient i with respect to day k
N_{i}^{Bl}	Number of days with NORMAL bleeding during the second heaviest episode during baseline of patient i
N_{ik}^{I}	Occurrence of NORMAL bleeding during the episode of interest of patient i with respect to day k
L_{i}^{Bl}	Number of days with LIGHT bleeding during the second heaviest episode during baseline of patient i
L_{ik}^{I}	Occurrence of LIGHT bleeding during the episode of interest of patient i with respect to day k
S_{ik}^{I}	Occurrence of SPOTTING during the episode of interest of patient i with respect to day k
Age_{i}	Age of patient i [years],

then we get the following equations for the estimation of the MBLV according (M1) based on the BI given in the diary of patient i at day k

if day k was a day with SPOTTING	if day k was a day with LIGHT bleeding
$MBLV_{Spotting\ ik} = \exp(2.5712$ $-0.1008\,Hb_{ik} + 0.002255\,Fe_{ik}$ $+0.0236\,H_{ik} - 0.02189\,H_{ik}^{-1} + 0.0863\,H_i^I$ $+0.01507\,N_{ik}^{MW} - 0.04875\,N_i^{Bl}$ $-0.04017\,L_i^{Bl} - 0.02261\,L_{ik}^I$ $-0.02386\,S_{ik}^I$ $+0.00535\,Age_i) - 1$	$MBLV_{light\ ik} = \exp(3.19185$ $-0.1008\,Hb_{ik} - 0.002133\,Fe_{ik}$ $+0.01273\,H_{ik} - 0.02221\,H_{ik}^{-1} - 0.2687\,H_i^I$ $+0.01507\,N_{ik}^{MW} - 0.04875\,N_i^{Bl}$ $-0.04017\,L_i^{Bl} + 0.05601\,L_{ik}^I$ $+0.10104\,S_{ik}^I$ $+0.01387\,Age_i) - 1$
**if day k was a day with NORMAL bleeding**	**if day k was a day with HEAVY bleeding**
$MBLV_{normal\ ik} = \exp(4.16355$ $-0.1008\,Hb_{ik} - 0.00327\,Fe_{ik}$ $-0.03281\,H_{ik} + 0.03253\,H_{ik}^{-1} - 0.4811\,H_i^I$ $+0.01507\,N_{ik}^{MW} - 0.04875\,N_i^{Bl}$ $-0.04017\,L_i^{Bl} + 0.14008\,L_{ik}^I$ $+0.02943\,S_{ik}^I$ $+0.02837\,Age_i) - 1$	$MBLV_{heavy\ ik} = \exp(5.01735$ $-0.1008\,Hb_{ik} - 0.00437\,Fe_{ik}$ $-0.05434\,H_{ik} + 0.04494\,H_{ik}^{-1} - 0.4811\,H_i^I$ $+0.01507\,N_{ik}^{MW} - 0.04875\,N_i^{Bl}$ $-0.04017\,L_i^{Bl} + 0.05601\,L_{ik}^I$ $-0.02386\,S_{ik}^I$ $+0.03026\,Age_i) - 1$

Table 1 Detailed description of model (M1)

Model (M1)					
Fixed Effects	BI	Estimate	Standard Error	LCL	UCL
Intercept		4.6027	0.2535	4.1055	5.0999
BI	Spotting	-2.4673	0.2738	-3.0044	-1.9302
BI	light	-1.9143	0.2138	-2.3336	-1.4950
BI	normal	-0.9745	0.1552	-1.2790	-0.6701
BI	heavy	0	.	.	.
Hb_{ik}		-0.1008	0.01042	-0.1212	-0.08034
Fe_{ik}		-0.00437	0.001062	-0.00645	-0.00229
Fe_{ik} *BI	Spotting	0.006625	0.001694	0.003305	0.009946
Fe_{ik} *BI	light	0.002237	0.001273	-0.00026	0.004733
Fe_{ik} *BI	normal	-0.00110	0.000973	-0.00301	0.000804
Fe_{ik} *BI	heavy	0	.	.	.
H_{ik}		-0.05434	0.01261	-0.07906	-0.02962
H_{ik} *BI	Spotting	0.07794	0.02101	0.03675	0.1191
H_{ik} *BI	light	0.06707	0.01720	0.03336	0.1008
H_{ik} *BI	normal	-0.02153	0.01771	-0.05623	0.01318
H_{ik} *BI	heavy	0	.	.	.
H_{ik}^{-1}		0.04494	0.01221	0.02102	0.06887
H_{ik}^{-1} *BI	Spotting	-0.06683	0.02065	-0.1073	-0.02635
H_{ik}^{-1} *BI	light	-0.06715	0.01743	-0.1013	-0.03297
H_{ik}^{-1} *BI	normal	0.01241	0.01756	-0.02200	0.04682
H_{ik}^{-1} *BI	heavy	0	.	.	.
H_i^I		-0.4811	0.09601	-0.6693	-0.2929
H_i^I *BI	Spotting	0.5674	0.1202	0.3317	0.8031
H_i^I *BI	light	0.2124	0.08462	0.04656	0.3783
H_i^I *BI	normal	0	.	.	.
H_i^I *BI	heavy	0	.	.	.
N_{ik}^{MW}		0.01507	0.004575	0.006104	0.02404
N_i^{Bl}		-0.04875	0.01562	-0.07936	-0.01814
L_i^{Bl}		-0.04017	0.009837	-0.05945	-0.02089
L_{ik}^I		0.05601	0.04037	-0.02311	0.1351
L_{ik}^I *BI	Spotting	-0.07862	0.05267	-0.1819	0.02462
L_{ik}^I *BI	light	0	.	.	.
L_{ik}^I *BI	normal	0.08407	0.05242	-0.01867	0.1868
L_{ik}^I *BI	heavy	0	.	.	.
S_{ik}^I		-0.02386	0.03135	-0.08532	0.03759
S_{ik}^I *BI	Spotting	0	.	.	.

Model (M1)					
Fixed Effects	BI	Estimate	Standard Error	LCL	UCL
S^{I}_{ik} *BI	light	0.1249	0.03947	0.04751	0.2022
S^{I}_{ik} *BI	normal	0.05329	0.03915	-0.02346	0.1300
S^{I}_{ik} *BI	heavy	0	.	.	.
Age_i		0.03026	0.004714	0.02102	0.03950
Age_i *BI	Spotting	-0.02491	0.006427	-0.03751	-0.01231
Age_i *BI	light	-0.01639	0.005060	-0.02630	-0.00647
Age_i *BI	normal	-0.00189	0.003664	-0.00908	0.005288
Age_i *BI	heavy	0	.	.	.

Table 2 Model (M1), fixed effects

Model (M1)	Patient		Residual Error	
Random Effects: Variance	Estimate	Standard Error	Estimate	Standard Error
Spotting	0.3238	0.03171	0.5478	0.01310
light	0.2658	0.02177	0.7411	0.01162
normal	0.3002	0.02514	0.7705	0.01437
heavy	0.3561	0.03092	0.4732	0.01240

Table 3 Model (M1), random effects

**Model (M2)**					
Fixed Effects	BI	Estimate	Standard Error	LCL	UCL
Intercept		3.9210	0.2332	3.4637	4.3783
BI	Spotting	-2.0222	0.2581	-2.5283	-1.5160
BI	light	-1.7589	0.2021	-2.1552	-1.3626
BI	normal	-0.9576	0.1553	-1.2621	-0.6530
BI	heavy	0	.	.	.
Hb_{ik}		-0.1011	0.01048	-0.1217	-0.08060
Fe_{ik}		-0.00452	0.001066	-0.00661	-0.00243
Fe_{ik} *BI	Spotting	0.006538	0.001699	0.003207	0.009869
Fe_{ik} *BI	light	0.002414	0.001273	-0.00008	0.004909
Fe_{ik} *BI	normal	-0.00098	0.000978	-0.00290	0.000937
Fe_{ik} *BI	heavy	0	.	.	.
H_{ik}		-0.04743	0.01243	-0.07179	-0.02307
H_{ik} *BI	Spotting	0.06983	0.02028	0.03009	0.1096
H_{ik} *BI	light	0.04835	0.01652	0.01598	0.08073
H_{ik} *BI	normal	-0.01346	0.01682	-0.04642	0.01950
H_{ik} *BI	heavy	0	.	.	.
N^I_{ik}		0.03505	0.01647	0.002759	0.06734
L^I_{ik}		0.06246	0.04039	-0.01671	0.1416
L^I_{ik} *BI	Spotting	-0.09965	0.05286	-0.2033	0.003948
L^I_{ik} *BI	light	0	.	.	.
L^I_{ik} *BI	normal	0.07293	0.05253	-0.03003	0.1759
L^I_{ik} *BI	heavy	0	.	.	.
S^I_{ik}		-0.02252	0.03140	-0.08407	0.03902
S^I_{ik} *BI	Spotting	0	.	.	.
S^I_{ik} *BI	light	0.1278	0.03951	0.05037	0.2052
S^I_{ik} *BI	normal	0.04854	0.03928	-0.02844	0.1255
S^I_{ik} *BI	heavy	0	.	.	.
Age_i		0.03285	0.004761	0.02352	0.04218
Age_i *BI	Spotting	-0.02394	0.006447	-0.03658	-0.01131
Age_i *BI	light	-0.01659	0.005044	-0.02648	-0.00670
Age_i *BI	normal	-0.00201	0.003683	-0.00923	0.005207
Age_i *BI	heavy	0	.	.	.

Table 4 Model (M2), fixed effects

**Model (M2)**		Patient		Residual Error
Random Effects: Variance	Estimate	Standard Error	Estimate	Standard Error
Spotting	0.3261	0.03189	0.5476	0.01310
light	0.2897	0.02338	0.7416	0.01163
normal	0.3337	0.02730	0.7739	0.01443
heavy	0.3761	0.03278	0.4746	0.01243

Table 5 Model (M2), random effects

**Model (M3)**					
Fixed Effects	BI	Estimate	Standard Error	LCL	UCL
Intercept		5.1672	0.1336	4.9052	5.4291
BI	Spotting	-2.9475	0.05658	-3.0585	-2.8365
BI	light	-2.2335	0.04349	-2.3188	-2.1482
BI	normal	-0.9132	0.03158	-0.9752	-0.8513
BI	heavy	0	.	.	.
Hb_{ik}		-0.1008	0.01055	-0.1215	-0.08009
Fe_{ik}		-0.00479	0.001086	-0.00691	-0.00266
Fe_{ik} *BI	Spotting	0.006537	0.001713	0.003179	0.009895
Fe_{ik} *BI	light	0.002459	0.001286	-0.00006	0.004979
Fe_{ik} *BI	normal	-0.00080	0.000980	-0.00272	0.001121
Fe_{ik} *BI	heavy	0	.	.	.

Table 6 Model (M3), fixed effects

**Model (M3)**	Patient		Residual Error	
Random Effects: Variance	Estimate	Standard Error	Estimate	Standard Error
Spotting	0.3314	0.03244	0.5484	0.01311
light	0.3074	0.02453	0.7421	0.01164
normal	0.3939	0.03133	0.7762	0.01446
heavy	0.4423	0.03730	0.4771	0.01249

Table 7 Model (M3), random effects

**Model (M4)**					
Fixed Effects	BI	Estimate	Standard Error	LCL	UCL
Intercept		4.4280	0.1244	4.1840	4.6721
BI	Spotting	-3.2645	0.1330	-3.5253	-3.0036
BI	light	-2.4975	0.09408	-2.6821	-2.3130
BI	normal	-1.0739	0.06642	-1.2042	-0.9437
BI	heavy	0	.	.	.
H_{ik}		-0.05295	0.01258	-0.07760	-0.02829
H_{ik} *BI	Spotting	0.08038	0.02096	0.03929	0.1215
H_{ik} *BI	light	0.07200	0.01719	0.03830	0.1057
H_{ik} *BI	normal	-0.02199	0.01771	-0.05671	0.01273
H_{ik} *BI	heavy	0	.	.	.
H_{ik}^{-1}		0.04963	0.01225	0.02561	0.07365
H_{ik}^{-1} *BI	Spotting	-0.06633	0.02067	-0.1068	-0.02582
H_{ik}^{-1} *BI	light	-0.06457	0.01747	-0.09882	-0.03033
H_{ik}^{-1} *BI	normal	0.01485	0.01764	-0.01972	0.04943
H_{ik}^{-1} *BI	heavy	0	.	.	.
H_{i}^{I}		-0.4375	0.1074	-0.6480	-0.2270
H_{i}^{I} *BI	Spotting	0.5229	0.1269	0.2742	0.7717
H_{i}^{I} *BI	light	0.1898	0.08808	0.01713	0.3624
H_{i}^{I} *BI	normal	0	.	.	.
H_{i}^{I} *BI	heavy	0	.	.	.
N_{ik}^{MW}		0.01856	0.004583	0.009574	0.02754
N_{i}^{Bl}		-0.05796	0.01714	-0.09156	-0.02435
L_{i}^{Bl}		-0.04800	0.01089	-0.06934	-0.02666
L_{ik}^{I}		0.05539	0.04050	-0.02400	0.1348
L_{ik}^{I} *BI	Spotting	-0.07872	0.05275	-0.1821	0.02466
L_{ik}^{I} *BI	light	0	.	.	.
L_{ik}^{I} *BI	normal	0.07913	0.05259	-0.02394	0.1822
L_{ik}^{I} *BI	heavy	0	.	.	.
S_{ik}^{I}		-0.02250	0.03148	-0.08421	0.03921
S_{ik}^{I} *BI	Spotting	0	.	.	.
S_{ik}^{I} *BI	light	0.1290	0.03957	0.05147	0.2066
S_{ik}^{I} *BI	normal	0.05629	0.03932	-0.02078	0.1334
S_{ik}^{I} *BI	heavy	0	.	.	.

Table 8 Model (M4), fixed effects

**Model (M4)**	Patient		Residual Error	
Random Effects: Variance	Estimate	Standard Error	Estimate	Standard Error
Spotting	0.3623	0.03455	0.5458	0.01303
light	0.3136	0.02489	0.7437	0.01166
normal	0.3983	0.03193	0.7727	0.01441
heavy	0.4545	0.03809	0.4747	0.01244

Table 9 Model (M4), random effects

**Model (M5)**					
Fixed Effects	BI	Estimate	Standard Error	LCL	UCL
Intercept		2.5439	0.1977	2.1561	2.9317
BI	Spotting	-1.8872	0.2594	-2.3959	-1.3785
BI	light	-1.5478	0.2019	-1.9438	-1.1517
BI	normal	-0.8546	0.1457	-1.1403	-0.5689
BI	heavy	0	.	.	.
Age_i		0.03411	0.005078	0.02416	0.04406
Age_i *BI	Spotting	-0.02476	0.006677	-0.03785	-0.01167
Age_i *BI	light	-0.01702	0.005185	-0.02718	-0.00686
Age_i *BI	normal	-0.00203	0.003729	-0.00934	0.005280
Age_i *BI	heavy	0	.	.	.

Table 10 Model (M5), fixed effects

**Model (M5)**	Patient		Residual Error	
Random Effects: Variance	Estimate	Standard Error	Estimate	Standard Error
Spotting	0.3592	0.03418	0.5465	0.01306
light	0.3335	0.02618	0.7448	0.01168
normal	0.4059	0.03203	0.7794	0.01453
heavy	0.4431	0.03755	0.4789	0.01255

Table 11 Model (M5), random effects
